# The Widespread Prevalence and Functional Significance of Silk-Like Structural Proteins in Metazoan Biological Materials

**DOI:** 10.1371/journal.pone.0159128

**Published:** 2016-07-14

**Authors:** Carmel McDougall, Ben J. Woodcroft, Bernard M. Degnan

**Affiliations:** 1 School of Biological Sciences, The University of Queensland, Brisbane, Queensland, Australia; 2 Australian Centre for Ecogenomics, The University of Queensland, Brisbane, Queensland, Australia; UMR-S1134, INSERM, Université Paris Diderot, INTS, FRANCE

## Abstract

In nature, numerous mechanisms have evolved by which organisms fabricate biological structures with an impressive array of physical characteristics. Some examples of metazoan biological materials include the highly elastic byssal threads by which bivalves attach themselves to rocks, biomineralized structures that form the skeletons of various animals, and spider silks that are renowned for their exceptional strength and elasticity. The remarkable properties of silks, which are perhaps the best studied biological materials, are the result of the highly repetitive, modular, and biased amino acid composition of the proteins that compose them. Interestingly, similar levels of modularity/repetitiveness and similar bias in amino acid compositions have been reported in proteins that are components of structural materials in other organisms, however the exact nature and extent of this similarity, and its functional and evolutionary relevance, is unknown. Here, we investigate this similarity and use sequence features common to silks and other known structural proteins to develop a bioinformatics-based method to identify similar proteins from large-scale transcriptome and whole-genome datasets. We show that a large number of proteins identified using this method have roles in biological material formation throughout the animal kingdom. Despite the similarity in sequence characteristics, most of the silk-like structural proteins (SLSPs) identified in this study appear to have evolved independently and are restricted to a particular animal lineage. Although the exact function of many of these SLSPs is unknown, the apparent independent evolution of proteins with similar sequence characteristics in divergent lineages suggests that these features are important for the assembly of biological materials. The identification of these characteristics enable the generation of testable hypotheses regarding the mechanisms by which these proteins assemble and direct the construction of biological materials with diverse morphologies. The SilkSlider predictor software developed here is available at https://github.com/wwood/SilkSlider.

## Introduction

Animals produce a diverse array of materials to aid with the multiple functions of life, including support, defence, feeding and reproduction. These structures are made from substances produced by the animal itself, which thus are ultimately encoded and/or regulated by the genome, sometimes with the inclusion of inorganic elements (e.g., calcium carbonate in the skeletons of many invertebrates). Their expression is precisely controlled at the nanoscale to produce structures with outstanding mechanical properties, such as silk, shells, bones, teeth, and hair, however the process by which this control is achieved is not yet fully understood. These biological materials are the inspiration for materials scientists, who are yet to fully emulate the properties of these substances, or to generate them at ambient temperatures and atmospheric pressure.

Arguably the best-studied biological material is silk, a fibre produced by a number of arthropods including spiders and silkworms that possesses remarkable properties including high elasticity and strength [1, 2]. Underlying the exceptional properties of silk fibroins are a set of extraordinarily large proteins with a modular, repetitive design. The repetitive regions are made up of distinct protein motifs including poly-A or poly-GA stretches, GPGXX/GPGQQ repeats and collagen-like GGX repeats (A = alanine, G = glycine, Q = glutamine, X = alanine, serine, valine, tyrosine or threonine) [3–6] that produce a modular protein consisting of crystalline domains interspersed with amorphous regions [1, 5, 7]. These high-performance fibres are utilized for essential biological processes such as reproduction and feeding, and are critical for the success of the organisms that produce them. The different types of silks produced by these animals are finely tuned at the molecular level for their particular purpose. For example, spider flagelliform silks,which comprise the capture spiral of the web, are highly elastic but have lower tensile strength due to the inclusion of proline residues within the typical glycine-rich repeats, whereas major ampullate silks, which form the framework of the web, are much stronger but less elastic due to the inclusion of increased poly-A and GGX repeats [3]. Therefore, the amino acid content and arrangement–both of which are mutable and therefore under natural selection—directly influences the physical properties of the protein.

Interestingly, the sequence features that are critical for the function of spider silks can also be found in proteins that are core components of tough, extracellular structures in other organisms. Proteins with this architecture have been described from mollusc shells [8–11], mussel byssus [12–15], lamprey cartilage [16], scallop hinge ligaments [17], polychaete tube cement [18], carp fertilisation envelopes [19], trematode eggshells [20, 21], human epidermal cell envelopes [22], cnidarian nematocysts [23] and even in plant cell walls [24, 25]. These proteins exhibit low sequence complexity, possessing single amino-acid tracts or sequence repeats of differing lengths [26–29]. They also often display modularity, containing one or more repetitive, low-complexity regions interspersed with other functional domains [13, 30, 31]. The practice of describing a non-silk structural protein as ‘silk-like’, based on these characteristics, is now common in the literature, despite the lack of primary sequence homology between these sequences and silk proteins. The term has been used to describe proteins with low-complexity glycine-rich regions, poly-alanine repeats, or both [11–15, 19, 23, 32–34], thus the true nature and extent of the proposed similarity remains undefined.

It is not clear whether the silk-like proteins described in the literature perform similar functions within the biological materials they form, although some insights can be garnered from a number of these proteins that have been the subject of biochemical and physical analyses due to their unusual mechanical properties and relevance for biomaterial design. Mussel byssal threads are the means by which these molluscs adhere to rocks against heavy wave action. They are primarily composed of preCol proteins, which are highly modular in nature and contain a central collagen domain [15]. However, the performance of byssal fibres significantly outperforms that of collagen itself, purportedly due to histidine-rich domains thought to mediate cross-linking between the proteins, poly-alanine rich domains that stiffen the fibre by the formation of crystalline beta sheets, and amorphous glycine-rich flanking domains which absorb stress and assist refolding of the protein once load is released [13]. Similarly, a number of silk-like proteins have been described from the organic matrix of molluscan shells [10, 11, 31, 35, 36]. These proteins also possess glycine-rich and/or poly-alanine rich regions, are localised within the organic matrix that surrounds the calcium carbonate tablets (possibly in the form of an amorphous gel) [32, 33, 37], and are thought to contribute to the strength, elasticity, and fracture toughness of the shell by absorbing strain applied to the shell that would otherwise cause it to crack [31, 38]. Therefore, these silk-like proteins possess sequence characteristics that increase the strength and elasticity of the materials which they form, and have the propensity to be amorphous in nature (notably, silk fibroins exist in a hydrated, disordered state within silk glands prior to spinning [39]). Interestingly, silk fibroins have been found to induce and regulate the mineralisation of CaCO_3_ and hydroxyapatite *in vivo* [40–43], providing further evidence that the similarities in amino acid sequences between silks and biomineralization proteins may be functionally significant.

The description of a number of silk-like proteins with functions in the production of biological materials raises a number of questions. First, exactly which sequence features (modularity, repetitiveness, poly-alanine motifs, and/or glycine-rich regions) contribute to this similarity, and how widespread is it across metazoan taxa and the materials they produce? Second, is the similarity within these sequences due to descent from an ancestral (presumably structural) protein, or have similar proteins arisen multiple times throughout metazoan evolution? And, finally, given the likely conserved functions of these proteins, can careful characterisation of sequence similarity reveal how the advanced mechanical properties of these biological materials are dictated by the sequence of the proteins that comprise them?

To answer these questions, we set out to systematically characterise these proteins and assess how widely they are distributed in metazoans that fabricate external biological materials. To do so, we identified defining sequence characteristics of proteins with silk-like or glycine-rich repeats that are known to contribute to tough, extracellular structures. We then used these sequence characteristics to develop a bioinformatic predictor for these proteins, which we named SilkSlider. This predictor was then used to survey the transcriptomes and genomes of a range of metazoan species that produce a diversity of biological materials. Using this method we identified genes encoding proteins with silk-like characteristics, which we call silk-like structural proteins (SLSPs), that are known components of biological materials in cnidarians, arthropods, nematodes, molluscs, echinoderms and chordates, as well as a large number of uncharacterized proteins from these taxa as well as from poriferans and annelids. To determine whether these uncharacterised proteins potentially represent hitherto unknown components of biological materials, we assessed their likely function in two distantly-related animals that produce well-studied biological materials, the abalone (a mollusc), and the sea urchin (an echinoderm), and found that a high proportion of predicted genes are associated with the production of shell or spicules, respectively. Our results indicate that the presence of SLSPs is widespread within biological materials produced by disparate metazoans. Interestingly, the genes encoding these proteins appear to have evolved multiple times independently in a number of lineages. The recurrent evolution of proteins with similar traits indicates that they perform common functions within biological materials, and that common principles underlie the formation of widely divergent biologically produced structures.

## Materials and Methods

### Predictor development

A literature survey identified 38 full-length biological material-related proteins that have either been described as silk fibroin-like or as glycine-rich. These sequences formed the ‘silk-like’ training dataset. A second dataset containing 100 secreted non-silk-like sequences formed the ‘non-silk-like’ training dataset (S1 Table, signal sequences were removed prior to analysis). Predictors based upon a) total percent glycine, b) total percent disorder (calculated using ESpritz [44], NMR prediction type), and c) percent glycine within a given window size (implemented via a custom script gly_sliding_window.rb now incorporated into the mature SilkSlider predictor), were tested for performance using ROC curves implemented R [45] using the program ROCR [46].

To test the predictor for its efficacy in identifying SLSPs, a list of known silk-like proteins from *Bombyx mori* was assembled from the literature (S2 Table, these sequences were excluded from the training dataset). To create a sequence database for the silkworm, all *B*. *mori* sequences were downloaded from the NCBI protein database (accessed 07 January 2014). Sequences lacking a N-terminal methionine were removed, resulting in a dataset of 19780 sequences. Using this dataset, the predictor was able to identify all 12 known *B*. *mori* silk proteins. For classification purposes, proteins with identical sequences were merged into one entry. For the purposes of reproducibility, the script gly_sliding_window.rb is available on github [47].

### Sequence analysis pipeline

Transcriptome and whole genome protein datasets were downloaded from publicly available databases (S3 Table), from a number of organisms including human, chicken, sea urchin, pearl oyster, abalone, polychaete, nematode, beetle, anemone, coral, placozoan and sponge. For transcriptomes, raw sequencing reads were assembled by de novo assembly using the CLC genomics workbench or the CAP3 program [48]. Assembled sequences were clustered using CD-HIT-EST [49] with default settings, and open reading frames (ORFs) and translations were determined using a custom Ruby script [50]. ORFs that were lacking an N-terminal methionine were removed from the analysis. N-terminal complete ORFs and whole genome protein datasets were then passed through a bioinformatic pipeline that 1) removed sequences that did not have a signal peptide using the program SignalP v 4.0 [51], 2) removed the signal peptide from remaining sequences using SignalP v 4.0, 3) removed sequences that were predicted to have a transmembrane domain by TMHMM v 2.0 [52], 4) used a sliding window algorithm to identify sequences containing at least 25% glycine within an 80 amino acid window. A software package implementing the above pipeline building upon BioRuby [53], and incorporating self-contained biogems [54] for using SignalP and TMHMM2, is available on github [55]. Splice variants were identified by referral to the corresponding genomic loci, where possible, and eliminated from the analysis. A BLASTP search using the NR protein database at NCBI was performed and the top hit (and top informative hit, if the top hit was to a predicted or hypothetical protein) recorded for all predicted silk-like proteins.

After BLASTP searches, each sequence was then allocated to a functional category using the Uniprot knowledgebase and literature searches where required. Categories and the criteria for classification are as follows: 1) ‘Known biological material protein’–protein has high similarity across the whole length to a protein that has been reported to be involved in biological material formation in an animal within the same phylum; 2) ‘Likely biological material protein’–protein has high similarity across the whole length to a protein that has been reported to be involved in biomaterial formation in a distantly-related organism, or to a protein that has been hypothesized to be involved in biological material formation; 3) ‘Collagen-like’–protein has high similarity to a characterized collagen, these are treated as a class of biological material-related protein; 4) ‘ECM related’–protein has high similarity across the whole length to a protein that has a known function in the extracellular matrix (ECM), these may play structural roles within the ECM and should not necessarily be treated as false hits; 5) ‘Known/likely false’–proteins with high similarity to characterized proteins that are known or likely to have roles that are not involved with biological material formation; and 6) ‘Uncharacterized’–proteins that have no similarity to other proteins in the database, or are similar to proteins for which the function is unknown. Proteins with low (E-value of e^-20^ or higher) similarity to proteins from distantly related organisms (e.g., bacteria) were also classed as ‘uncharacterized’. Sequences that were clear homologues of well characterized non-secreted or transmembrane proteins were assumed to be the result of incorrect model prediction, and were removed from the analysis. All proteins identified by the predictor and their classifications are presented in S1 Dataset.

### Material sources and *in situ* hybridization

*In situ* hybridization was performed to determine whether previously undescribed genes identified by the predictor are expressed in tissues consistent with roles in biological material formation. The tropical abalone *Haliotis asinina* were spawned and cultured as previously described [56]. Competent larvae were induced to settle on biofilmed plates, juveniles were fed on algae growing naturally in the settlement tanks. Fixation and decalcification of juveniles was performed as previously described [57]. Probes were synthesized using DIG RNA labelling mix (Roche) according to the manufacturer’s instructions from PCR products generated from the following primers (5’ to 3’) that were cloned into the pGEM-T Easy vector (Promega):

HasCL10Con2-Fwd TGCTTACGATCAAGCCAGTG;

HasCL10Con2-Rev CAGAAGCTGATGCACGGATA;

HasGRBP-Fwd TTCTGAAAGATGGCGGAAGT; and

HasGRBP-Rev AAGTTCATCTGCACGGCTCT. Hybridizations were performed as previously described [58, 59], with 50 μg/ml proteinase K digestion at 37°C for 15 minutes and a hybridization temperature of 60°C.

Fixed embryos and larvae of the sea urchin *Strongylocentrotus purpuratus* were kindly provided by Fred Wilt. Probes were synthesized as above using PCR products generated from the following primers (5’ to 3’):

SpuCara7LA-Fwd CAACTCAGCTCCAACGACAA;

SpuCara7LA-Rev GGCAGACAAAAGCCATGATT;

SpuSM30E-Fwd CAACAACCAAGATGGGCTTT;

SpuSM30E-Rev CTGTATTTGATGGGCGACCT;

SpuSM30B/C-Fwd ATTGGCTTTGGCCTCTTTCT;

SpuSM30B/C-Rev AGGGATGGTACTCGCAGATG;

Spuhbn-Fwd TGAGAAATCCAATCGGGAAG; and

Spuhbn-Rev GATGCAGTTGGAATGTGGTG. Hybridizations were performed according to previously described methods [60], with 5 μg/ml proteinase K digestion at room temperature for 10 minutes and a hybridization temperature of 50°C. After staining, specimens were dehydrated in an ethanol series and cleared in a 2:1 solution of benzyl benzoate and benzyl alcohol. [58–60] For both species, negative controls (no probe added) were performed and showed no staining, and positive controls produced the expected expression patterns (S1 Fig). No ethics approval was required for this study.

### Peptide match

To determine whether proteins identified by the predictor can be found within biological materials, all silk-like proteins from *S*. *purpuratus* were assembled into a database and queried for peptide sequences previously isolated from spicule, test, spines and teeth [61–63] using a local installation of Peptide Match [64]. Default settings were used, and leucine and isoleucine were not treated as equivalent.

### Rapid amplification of cDNA ends (RACE)

To obtain the full-length sequence of the abalone P0020O08 (*Has-GRBP*) gene (GenBank GT276076.1), RACE-ready cDNA libraries were constructed from mixed larval and juvenile *H*. *asinina* total RNA. Reactions were performed using the BD SMART RACE cDNA Amplification Kit (Clontech) as per the manufacturer’s instructions, using the following primer:

HasGRBP-3: 5’ AGCCGAACTGGATGACAGATGCAAG. The resulting product was cloned into pGEM-T Easy vector (as above), and sequenced using vector primers and the internal primers HasGRBP-7: 5’ ATGGCTGCCCAAGGATTAAC and HasGRBP-9: 5’ GTCACGTTAACCCCAGTCGT. The resulting full-length sequence has been deposited to GenBank (accession number KJ842084).

### All versus all BLAST

To investigate sequence similarities between SLSPs identified from different taxa, BLASTP searches (with glycine residues masked) were conducted using these sequences as queries against a BLAST database constructed from all predicted SLSP sequences (e-value cutoff of 1xE-20). BLAST searches were performed using a local installation of ncbi-blast-2.2.30+ [65].

## Results

### Predictive characteristics of silk-like proteins

**Fig 1:**
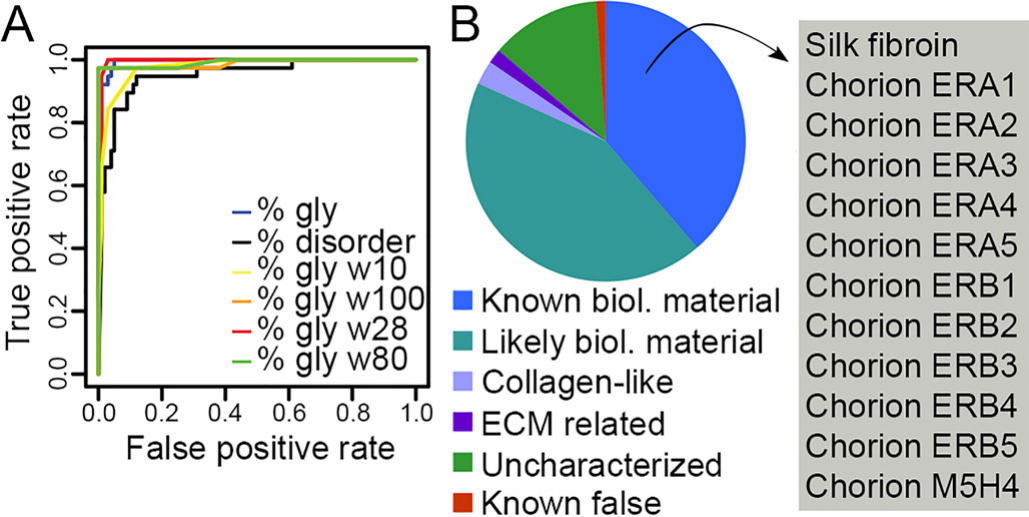
A. ROC curves displaying the performance of different predictors. B. Categorisation of silk-like proteins identified in the *B*. *mori* dataset. The pie chart shows division of the 178 predicted silk-like proteins into the categories indicated in the legend. ‘Known’ and ‘likely biological material’ categories refer to proteins that are known or likely to have a role in biological material formation. All 12 known *B*. *mori* silk-like sequences found in the literature (box) were identified by the predictor.

To explore the relationship between glycine content and the silk-like proteins commonly found in biological materials, we assessed whether particular sequence features could accurately classify silk-like and non-silk like genes within a test dataset comprising 38 known silk-like structural proteins and 100 randomly selected secreted proteins (S1 Table). We found that using overall glycine content as a criteria performed very well, with the resulting receiver operating characteristic (ROC) curve displaying a very high true positive rate to false positive rate ratio with an Area Under the ROC Curve (AUC) of 0.995 (Fig 1A, blue line; perfect assignment is equal to an AUC of 1). As protein disorder has also been found to be an important characteristic of biological material-related proteins [66, 67], and because disorder can be conferred by a high glycine content [68], we assessed whether overall protein disorder would be an equal (or better) predictor of silk-like proteins. We found that predictions based on disorder performed well, but more poorly than those based on glycine content alone (Fig 1A, black line, AUC = 0.951).

Many silk-like proteins are modular in nature, combining glycine-rich regions with other functional domains [13, 29–31]. We therefore tested whether incorporating a sliding-window algorithm [69] would improve the performance of the predictor. Window sizes of 10 (Fig 1A, yellow line, AUC = 0.976) to 100 (Fig 1A, orange line, AUC = 0.989) amino acids were tested, with windows above 27 amino acids improving prediction accuracy. We found that a window size of 28 (Fig 1A, red line, AUC = 0.995) enabled 100% accurate prediction of true positives and a low false positive rate, and that a window size of 80 (Fig 1A, green line, AUC = 0.992) allowed a high true positive rate with a 0% false positive rate. The data used in the testing can be found in S2 Dataset.

From these analyses we developed a tool to predict silk-like, structural proteins from large sequence datasets. The pipeline first predicts which protein sequences likely produce secreted products, and then uses a sliding-window algorithm to identify the secreted proteins which have a domain of 80 amino acids that contains at least 25% glycine residues (the threshold providing the optimum distinction between silk-like and non-silk-like genes, as determined from the ROC curves). This window size was chosen to minimize false positives, however the parameters can be altered to maximize the identification of true positives by using a window size of 28 and a percent glycine cutoff of 35. We have called our prediction tool ‘SilkSlider’.

### Test of SilkSlider accuracy

To assess the efficacy of our predictor, we tested it on the silkworm (*Bombyx mori*), which has several previously identified glycine-repeat rich proteins known to form tough extracellular structures (Fig 1B). The most obvious and well-studied of these is the incredibly strong silk fibroin heavy chain [6, 70]; a number of structural protein components of the silkworm egg chorion have also been identified [71–73]. SilkSlider was applied to *B*. *mori* proteins present in the NCBI protein database and successfully identified all 12 known silk-like sequences (Fig 1B, S2 Table).

In total, SilkSlider identified 178 SLSPs in the *B*. *mori* protein database (Fig 1B), including coding sequence for 74 proteins with known biological material-related roles (mostly cuticle proteins; the silk-like nature of some cuticle proteins has been previously discussed in the literature [74, 75]), 71 proteins that likely have a biological material-related role (based on similarity to other proteins), 5 collagen-like proteins, 3 proteins with roles in the extracellular matrix (ECM), and 25 uncharacterized proteins. Importantly, none of the identified proteins are known to have non-biological material roles. Therefore the predictor performs well in detecting previously characterized proteins that are known or likely to be components of biological structures/materials, as well as unknown proteins that may also fulfil this role.

### Survey of silk-like proteins across the animal kingdom

**Fig 2:**
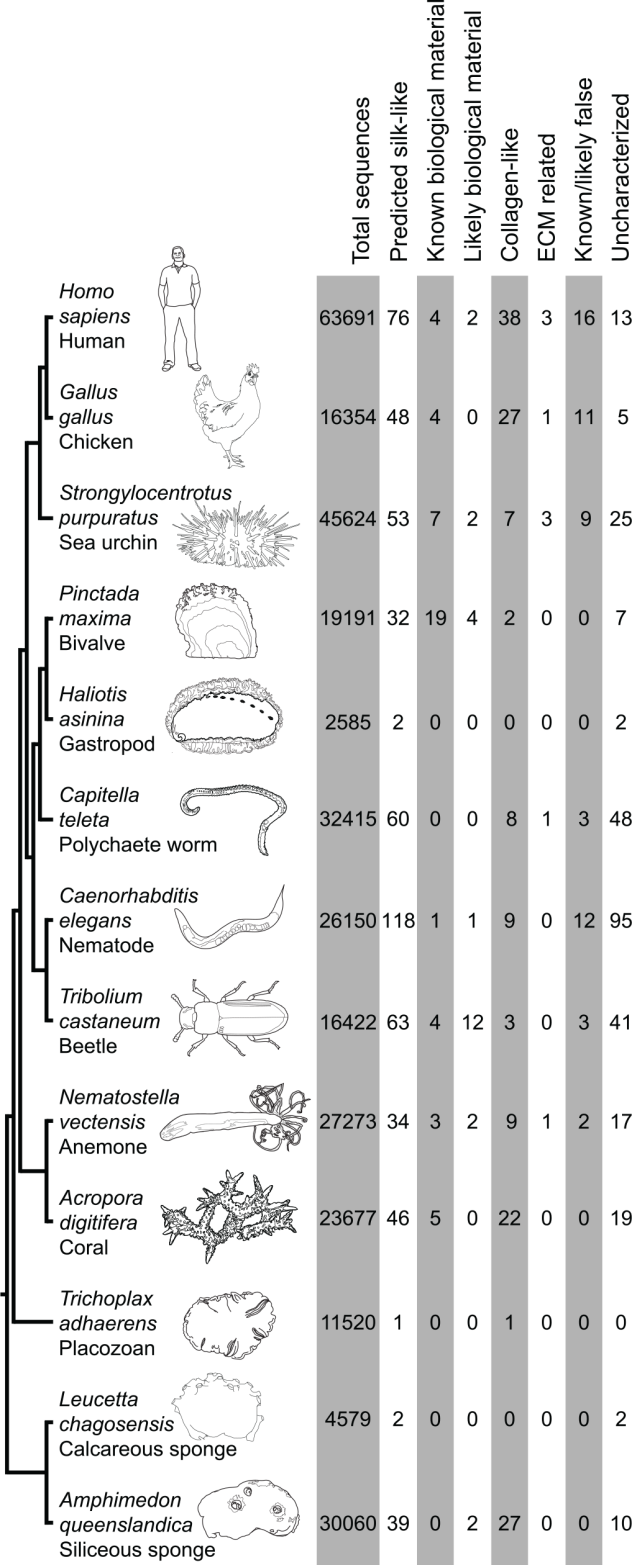
Results of survey of silk-like proteins in the Metazoa. Dendrogram on the left indicates currently accepted phylogenetic relationships of investigated taxa. Columns on the right indicate the total number of sequences evaluated by the predictor, the total number of predicted silk-like proteins, and the categorisation of identified silk-like proteins, for each taxon.

To assess whether the same principles can be applied in other species, we used SilkSlider on assembled transcriptome datasets and predicted proteins from whole genomes from a wide range of animals (Fig 2, S1 Dataset). These animals produce a range of tough, extracellular structures and it is unknown whether the characteristics that can be used to identify proteins involved in silk, cuticle and chorion production in the silkworm can also be used to identify proteins involved in biological material production in other organisms.

As expected, SilkSlider identified cuticle proteins in the beetle and nematode genomes, and we found that the predictor also identified other known biological material-associated proteins in most datasets (Fig 2, S1 Dataset). For instance, otolin, a key structural protein of the inner ear [76], was identified from vertebrate genomes and, interestingly, potentially in the sea urchin. From coral and anemone genomes SilkSlider identified nematogalectin, a key structural protein in nematocyst tubules [77], and minicollagen, a structural component of nematocyst capsules [78]. A number of identified proteins were biomineral-related, such as the shematrin and KRMP proteins from pearl oyster shells [10, 29, 79], spicule matrix proteins from sea urchin larval spicules [80], and ovocleidin from chicken eggshells [81]. SilkSlider also identified numerous collagen-like and ECM related proteins from the datasets. A high proportion of the proteins identified in most taxa were classified as uncharacterized, with no significant identity to sequences in the NCBI database (other than those classified as ‘hypothetical’ or ‘predicted’). In general, the number of genes identified by SilkSlider with known non-biological material related roles was low, however the nematode, sea urchin and human datasets produced a higher number of false positives. This was due to the lineage-specific expansion of a hedgehog-related family in the nematode [82] and an immunity-related gene family in the sea urchin [83], each of which has a glycine-rich region. Interestingly, a number of genes involved in human innate immunity also have glycine-rich sequences and were identified by the predictor, including C1q proteins of the complement system [84].

We further tested SilkSlider on the whole genome of the placozoan, *Trichoplax adherens*, an animal that appears to lack any tough, extracellular structures [85]. We would therefore expect the predictor to identify few, if any, silk-like proteins. As expected, SilkSlider identified a sole protein (likely a type IV collagen) [Genbank:XP_002116296] from the whole genome data.

### Predicted silk-like proteins are involved in biological material formation

To support the hypothesis that previously uncharacterised sequences identified by the predictor have a role in biological material formation, we investigated these genes and the proteins they encode in the tropical abalone *Haliotis asinina* and the purple sea urchin *Strongylocentrotus purpuratus*. These taxa were chosen because 1) they possess obvious biomineralized biological materials (the abalone shell, and sea urchin spicules, spines, test and mouthparts) for which the underlying cellular basis is understood [59, 80, 86], 2) they represent taxa for which different levels of genomic resources are available, i.e., full genome sequence and extensive transcriptome data for the sea urchin, in contrast to a small transcriptome for the abalone, and 3) they have both been subject to proteomic analysis for the primary biomineralized structures they produce [61–63, 87].

**Fig 3:**
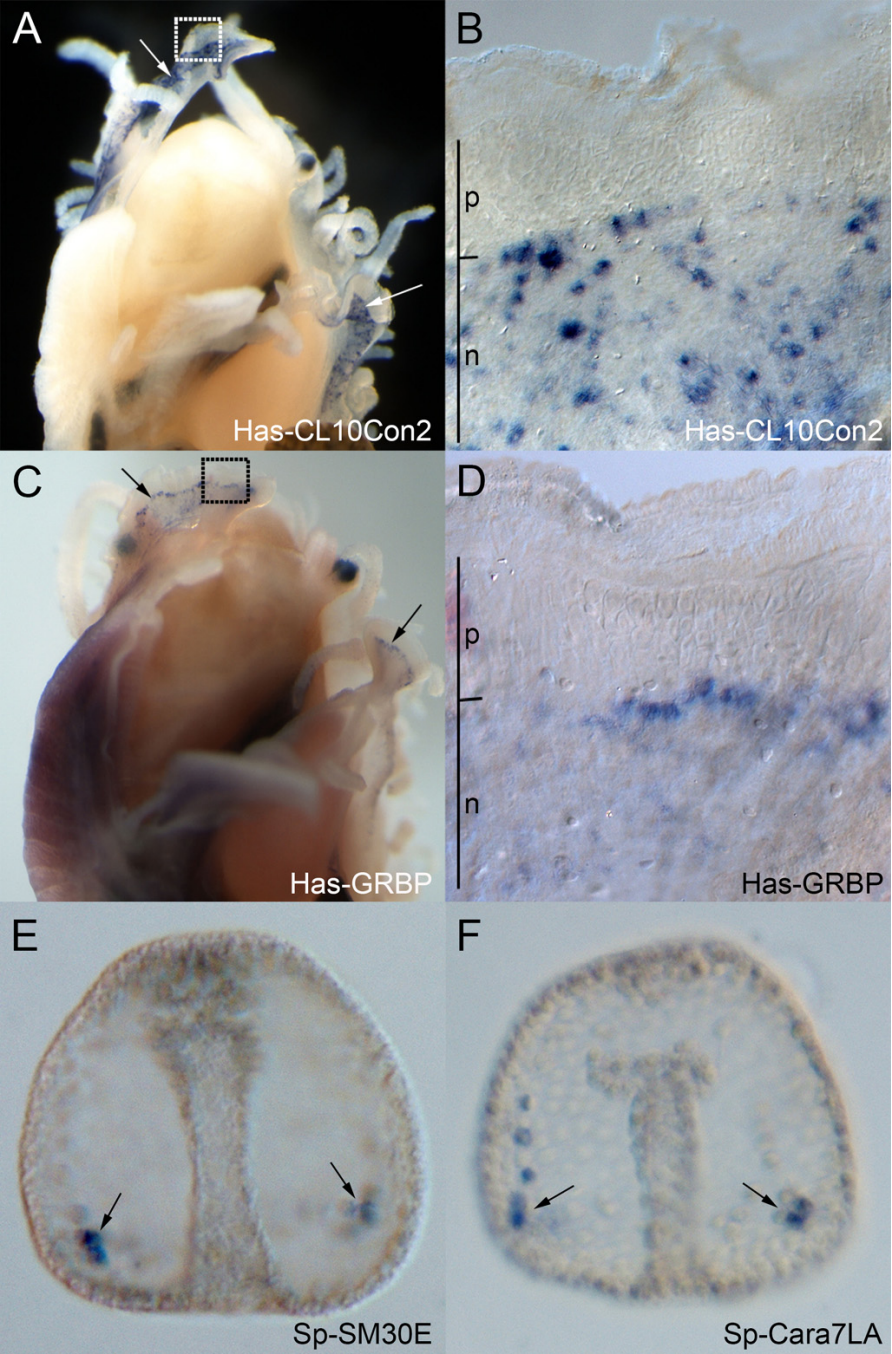
Spatial localization of genes encoding predicted silk-like proteins. Blue staining corresponds to cells expressing the gene. A. Dorsal view of juvenile *H*. *asinina*, removed from shell. *Has-CL10Con2* expression is restricted to the mantle (arrows). B. Expanded view of boxed area in ‘A’. *Has-CL10Con2* expression is in cells in the nacreous zone of the mantle. C. Dorsal view of juvenile *H*. *asinina*, removed from shell. *Has-GRBP* transcripts are localized to the mantle (arrows). D. Expanded view of boxed area in ‘C’. *Has-GRBP* expression is restricted to cells in the nacreous zone of the mantle, and is higher at the boundary between prismatic and nacreous zones. E. Late gastrula-stage (40 hours post fertilisation) *S*. *purpuratus* embryo. The sea urchin *SM30E* gene is expressed in PMCs (arrows). F. Late gastrula-stage (40 hours post fertilisation) *S*. *purpuratus* embryo. The sea urchin *Cara7LA* gene is expressed in PMCs (arrows). p, prismatic zone; n, nacreous zone.

SilkSlider identified two silk-like proteins in the abalone *H*. *asinina*, neither of which had any significant similarity to other sequences in the NCBI protein database (S1 Dataset). The most glycine-rich sequence, Has-CL10Contig2 [GenBank:EZ420619], possesses several different repetitive regions including a [GN]_n_ repeat and a [GGSGGSGFG] _n_ repeat. *In situ* hybridization revealed that the gene encoding this protein is restricted to the part of the mantle responsible for producing the nacreous (mother-of-pearl) shell layer (Fig 3A and 3B). The full sequence of the second silk-like sequence was determined by RACE [Genbank:KJ842084] and is less repetitive, possessing only short [GMGA] _n_ and [QQQV] _n_ repeats. Like *Has-CL10Contig2*, *in situ* hybridization analysis shows that this gene is expressed in the nacre-producing part of the mantle. However, there is clear up-regulation of gene expression in the boundary between nacreous and prismatic producing zones (Fig 3C and 3D), hence we named this sequence *Has-GRBP* (Glycine Rich Boundary Protein). The restricted expression of these genes to this mantle region indicates a high likelihood that the proteins are involved in the production of the shell; this is supported by the isolation of peptides corresponding to Has-CL10Contig2 from the shell in a proteomic study conducted on *H*. *asinina* [87].

The predictor identified 53 silk-like sequences from the sea urchin *S*. *purpuratus* genome, of which 9 were known or likely structural, 7 were collagen-like, 3 were related to the extracellular matrix (ECM), 12 were known or likely false, and 25 were uncharacterized (based on sequence homology, S1 Dataset). *In situ* hybridization of two of these genes, one a known component of the larval skeleton (*Sp-SM30E*), and the other an uncharacterized gene predicted to encode a novel carbonic anhydrase (*Sp-Cara7LA*), revealed that they are both expressed at post-gastrula stages in the primary mesenchyme cells (PMCs), which are the cells responsible for secreting larval spicules [80] (Fig 3E and 3F). *S*. *purpuratus* is a model species for invertebrate development, and the process of biomineralization, in particular, has been extensively studied. Large-scale proteomic analyses have been conducted on *S*. *purpuratus* calcified parts, which include the larval spicules and adult test, spines and teeth [61–63]. Additionally, a recent genome-wide analysis of components of the gene regulatory network of *S*. *purpuratus* identified genes that were differentially expressed in PMCs compared to the rest of the embryo, as well as genes for which expression levels were affected by knockdown of Alx1 and Ets1, key transcription factors controlling the skeletogenic program in this species [88]. The sequences of 20 of the predicted silk-like genes matched peptides identified in the biomineral proteomes and are therefore true components of these biomineralized structures, while 14 predicted sequences were also identified as being differentially expressed in PMCs or as being affected by Alx1/Ets1 knockdowns (6 were identified in both studies, see S1 Dataset). These validated sequences included those classified as ‘ECM’, ‘collagen-like’, ‘likely false’ and ‘known false’ based on sequence homology (S1 Dataset**)**. Therefore, many of the sequences identified by SilkSlider that were considered to be false positives, based on sequence similarity, may actually have roles in biological material formation, and the accuracy of SilkSlider is likely underestimated when assessed by sequence similarity-based annotation alone.

### Silk-like proteins have likely evolved independently in numerous animal lineages

The silk-like proteins identified in this study were used in an all-against-all BLASTP search (with glycine residues masked) to reveal whether any proteins exhibited sequence similarity between different taxa (S3 Dataset). Proteins with broad taxonomic distributions include collagens, fibrillin and SCO-spondin. Several proteins exhibited phyla-restricted distributions, including nematogalectin in cnidarians, several cuticle proteins in arthropods, and specific collagen types in vertebrates. A number of proteins of unknown function also shared similarity between different taxa. In total, only 22% of the proteins exhibited sequence similarity with a protein identified from another taxon, revealing a high level of novelty within SLSPs.

## Discussion

### Proteins containing glycine-rich domains are involved in biological material production in diverse metazoans

In this study, we have confirmed that many proteins involved in the production of biological materials possess similarity to silks, and that this similarity is primarily due to a high proportion of glycine in at least part of the protein. These SLSPs are widespread throughout metazoans, being found in the transcriptomes and genomes of all animals investigated here (although the predictor identified a single gene, collagen, in the placozoan *Trichoplax*; this organism does not appear to produce any tough, extracellular structures).

The predictor developed during the course of this study identified a number of proteins to which no functions have yet been ascribed. To determine whether they are potentially undescribed components of biological materials, we assessed the localization of expression of the genes corresponding to several of these proteins in two animals, the gastropod mollusc *H*. *asinina* and the sea urchin *S*. *purpuratus*. Two uncharacterized proteins were identified by the predictor in the abalone *H*. *asinina* as being silk-like, and both had expression patterns consistent with a structural role in the nacreous layer of the shell. *Sp-Cara7LA* is an uncharacterized *S*. *purpuratus* gene that encodes both glycine-rich and carbonic anhydrase domains, and is expressed in the PMCs, consistent with a role in biomineralization in the sea urchin. These functional predictions are further supported by the extraction and characterisation of Has-CL10Contig2 and Sp-Cara7LA proteins from the abalone shell [87] and sea urchin calcified structures [61–63], respectively. Sequences identified by the predictor that have not been detected in proteomic analyses of sea urchin calcified parts may be minor (and thus undetected) components of these structures, or be involved with the production of other biological materials within this animal.

### Recurrent evolution of SLSPs in animals

SLSPs can be found throughout the animal kingdom and are often components of structures that are morphological novelties for that particular taxon, such as the shells of molluscs, the nematocysts of cnidarians and the tests of sea urchins. It is therefore unlikely that they were inherited from a common ancestor, as there was no common precursor to these structures. The lack of primary sequence conservation between silk-like proteins (outside the presence of glycine-rich domains) also suggests that they arose independently multiple times. Consistent with this, very little similarity has been observed between shell-forming proteomes of various molluscs [27, 89], and the spicule matrix (SM) gene family, which is crucial for the formation of sea urchin larval spicules [90, 91], is completely absent from the genome of the closely-related hemichordates that also produce biomineralized structures [92]. The apparent convergent evolution of silk-like proteins in the production of biological materials suggests that high glycine content is functionally advantageous for this class of proteins.

SLSPs appear to fall into two broad classes: those that likely evolved from existing functional protein coding sequences and those that appear to have evolved *de novo*. Several important biomineralization genes, such as sea urchin *Cara7LA*, encode glycine-rich regions in combination with other domains with biomineralization-related roles. In Cara7LA, a glycine-rich domain is combined with a carbonic anhydrase domain, an arrangement also seen in pearl oyster nacrein proteins [93]. Similarly, some sea urchin SM family genes encode both C-type lectin [94] and glycine-rich domains. Nematogalectins, core components of the nematocyst tubule in cnidarians, combine glycine-rich and galectin domains [77]. The occurrence of glycine-rich domains in proteins that likely already had a function within biological materials is consistent with silk-like properties evolving in some genes that were already part of the biological material regulatory networks in these animals. On the other hand, the expression of numerous lineage-specific silk-like genes in animal tissues responsible for fabricating external structures, such as the *lysine (K)-rich mantle protein* (*KRMP*) and *shematrin* genes in pearl oysters [24], suggests that some silk-like genes evolved de novo and were subsequently incorporated into a role in the formation of these structures.

### Sequence similarities within SLSPs provide insight into the function of these proteins and the mechanism underlying the production of biological materials

The predictor developed in this study is able to detect proteins that are involved in biological material production from large-scale sequence databases, demonstrating a correlation between sequence characteristics and functional roles. The sequence characteristics found to be important for the identification of these proteins (i.e., common to this class of protein) are the possession of a signal peptide (most biological materials are extracellular) and a glycine-rich domain. The importance of glycine is likely because of the properties it confers to the secondary structure of the protein; glycine has smaller side-chains than other amino acids, and appropriate spacing of glycine residues in a sequence is critical for the formation of various ordered structures such as beta-sheets, coiled-coils and collagen-like triple helices, with the final secondary structure being determined by other amino acid residues within the sequence [95]. Proteins with these ordered structures are known to be important components of biological materials such as spider silks and mollusc shells [95, 96]. However not all proteins that are identified by the predictor, including some that are known components of biological materials, have the regular arrangement of glycine residues necessary for the generation of these secondary structures. High glycine content is also known to be important for the flexibility of disordered protein domains; such disordered proteins confer elastomeric properties to biological materials and are also known to be components of spider silks and mollusc shells [66, 68]. The glycine-rich domain common to these sequences could therefore facilitate the formation of either folded secondary structures or disorder within the proteins they are found in. It is possible that both of these configurations are important, given that SilkSlider outperforms disorder-based predictors in identifying proteins with biomaterial-related roles.

The ability to predict biological material-related proteins based upon primary sequence indicates that common mechanisms may underlie the fabrication of biological materials in different animals. It is possible that proteins with glycine-rich regions provide increased elasticity and toughness to the structures they form, as proposed for several SLSPs [13, 23, 31]. Alternatively, glycine-rich regions may be important for the assembly of the structures. Molluscan biomineralized materials, in particular, are thought to be constructed within a gel-like protein matrix, and the intrinsic disorder of the components is essential for the formation of the gel itself [32, 97]. Additionally, the amorphous, glycine-rich domains within mussel byssal threads are thought to facilitate the reformation of bonds after stress [13]. Additional clues may be provided by the apparent threshold of glycine content for SLSPs (25% glycine within an 80 residue window). Manipulation of the glycine content of SLSP-inspired peptides based upon this threshold and observation of the effects on the formation of biomaterials and their properties will reveal the functional relevance of these glycine-rich sequences.

## Conclusions

Nature is capable of producing materials that far exceed the current technical capabilities of humankind. In this study we reveal that a class of proteins, the SLSPs, are components of these materials in a wide range of taxa. These proteins can be defined as possessing a signal peptide and a domain in which at least a quarter of the residues are glycine. Despite these common features, these proteins have evolved numerous times independently in different lineages. The glycine-rich domains likely confer elasticity and toughness to the materials these proteins form, and/or facilitate their construction by the formation of amorphous gels. This research suggests that common principles underlie the construction of divergent biological materials, despite them being made from evolutionarily distinct proteins.

## Supporting Information

S1 Dataset Predicted silk-like sequences. Spreadsheet of silk-like sequences identified from each taxon in this study (each taxon on a separate sheet).
(XLSX)

S2 Dataset Raw data for ROC curve generation. Calculation of overall glycine percent, overall percent disorder, and maximum glycines by window size for all sequences within the training dataset.
(XLSX)

S3 Dataset Results of all-against-all similarity searches. Interphyla (sheet 1) and intraphyla (sheet 2) similarity found in predicted silk-like sequences.
(XLSX)

S1 Fig *In situ* hybridization controls. Figure showing results of control *in situ* hybridizations for *H*. *asinina* and *S*. *purpuratus*.
(DOCX)

S1 Table Silk-like and non-silk-like sequences used for predictor development. Table of sequences used for predictor development, including accession numbers.
(DOCX)

S2 Table Known *B*. *mori* silk-like proteins. Table of known silkworm silk-like proteins and their accession numbers.
(DOCX)

S3 Table Sources of sequence data used for analysis. Table with details of the data used for analyses, including websites and references.
(DOCX)
