# Supplementary material for: The Widespread Prevalence and Functional Significance of Silk-Like Structural Proteins in Metazoan Biological Materials
Source: PLoS One. 2016 Jul 14;11(7):e0159128. doi: 10.1371/journal.pone.0159128 (PMC4944945; doi:10.1371/journal.pone.0159128)
Supplement: S1 Fig — Figure showing results of control in situ hybridizations for H. asinina and S. purpuratus. (DOCX) [file pone.0159128.s004.docx]

**
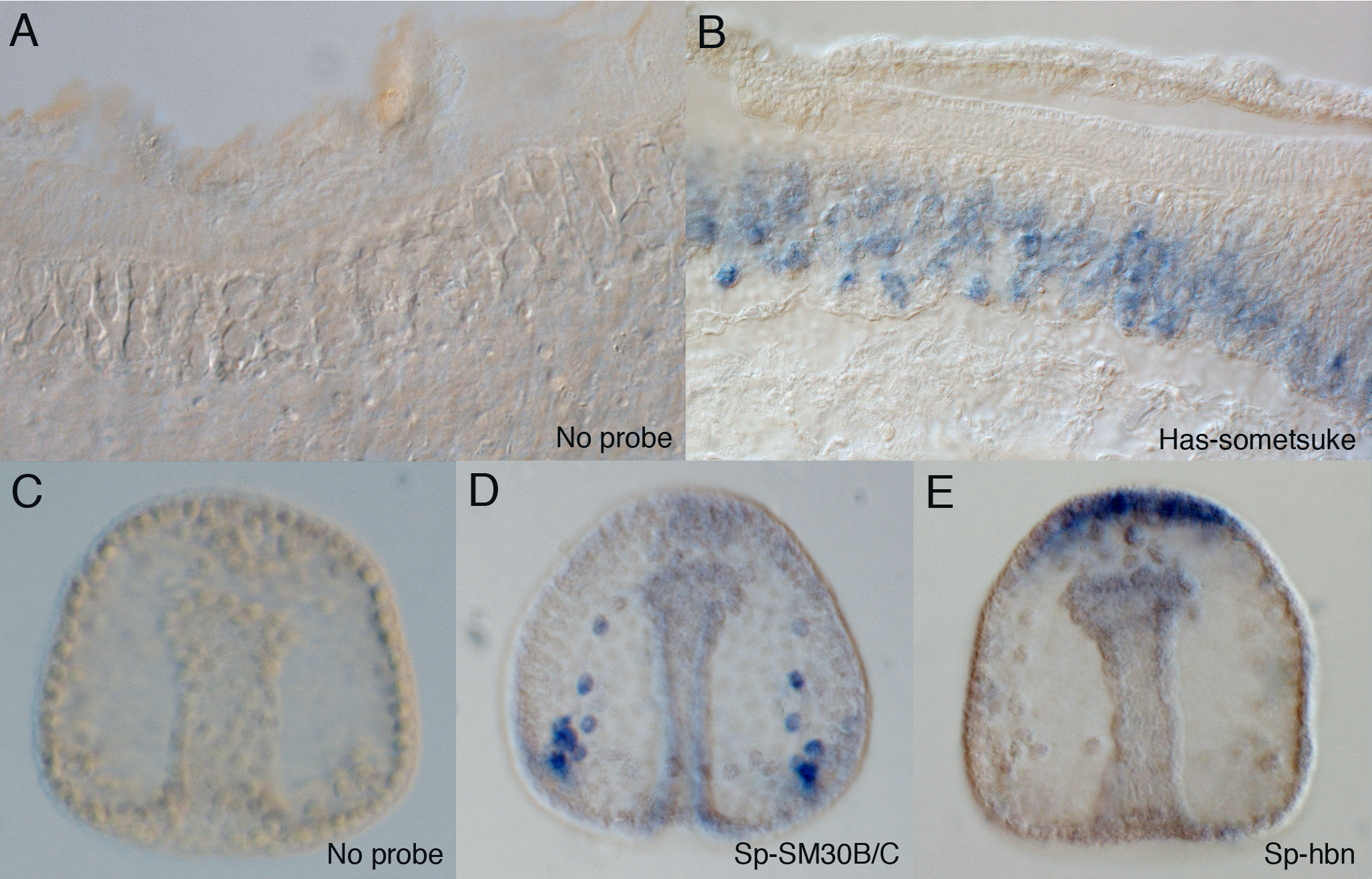
**

**S5 Fig. *In situ* hybridization controls.** Blue staining represents gene localization. A. Dorsal view of juvenile *H. asinina* mantle, no probe control. B. Dorsal view of juvenile *H. asinina* mantle, positive control. The gene investigated is *Has-sometsuke*, expressed in the prismatic zone of the mantle (6). C. 40 hours post fertilization (hpf) *S. purpuratus* embryo, no probe control. D. 40 hpf *S. purpuratus,* positive control. The gene investigated is *Sp-SM30B/C*, expressed in primary mesenchyme cells (8). E. 40 hpf *S. purpuratus*, off-target control. The gene investigated is *Sp-hbn*, expressed in the apical organ (9).
