# Supplementary material for: The Widespread Prevalence and Functional Significance of Silk-Like Structural Proteins in Metazoan Biological Materials
Source: PLoS One. 2016 Jul 14;11(7):e0159128. doi: 10.1371/journal.pone.0159128 (PMC4944945; doi:10.1371/journal.pone.0159128)
Supplement: S1 Table — Table of sequences used for predictor development, including accession numbers. (DOCX) [file pone.0159128.s005.docx]

**S1 Table: Silk-like and non-silk-like sequences used for predictor development**

|  | **Organism** | **Sequence name** | **Genbank accession** |
| --- | --- | --- | --- |
| **Silk-like** | *Antheraea pernyi* (Silkmoth) | Fibroin heavy chain | AAC32606.1 |
|  | *Latrodectus hesperus* (Spider) | Major ampullate spidroin 1 | ABR68856.1 |
|  | *Latrodectus hesperus* (Spider) | Major ampullate spidroin 2 | ABR68855.1 |
|  | *Cyprinus carpio* (Fish) | Fibroin-like substance 1 | AAG25716.1 |
|  | *Cyprinus carpio* (Fish) | Fibroin-like substance 3 | AAG25718.1 |
|  | *Mytilus edulis* (Mussel) | pre-Col-D | AAB96638.1 |
|  | *Mytilus californianus* (Mussel) | pre-Col-D | ABW90432.1 |
|  | *Mytilus californianus* (Mussel) | pre-Col-P | ABW90434.1 |
|  | *Mytilus galloprovincialis* (Mussel) | pre-Col-D | AAM34601.1 |
|  | *Mytilus galloprovincialis* (Mussel) | pre-Col-P | AAM34600.1 |
|  | *Mytilus edulis* (Mussel) | pre-Col-P | AAB80719.1 |
|  | *Mytilus californianus* (Mussel) | pre-Col-NG | ABW90433.1 |
|  | *Mytilus galloprovincialis* (Mussel) | pre-Col-NG | AAM34599.1 |
|  | *Argopecten irradians* (Scallop) | Abductin | AAB94680.1 |
|  | *Drosophila melanogaster* (Fly) | Ejaculatory bulb protein | AAD55736.1 |
|  | *Phragmatopoma californica* (Polychaete) | Cement protein 1 | AAY29115.1 |
|  | *Phragmatopoma californica* (Polychaete) | Cement protein 2 | AAY29116.1 |
|  | *Manduca sexta* (Moth) | Putative cuticle protein | AAF16693.1 |
|  | *Schistosoma mansoni* (Trematode) | Putative eggshell precursor | P06649.1 |
|  | *Opisthorchis viverrini* (Trematode) | Glycine-tyrosine rich eggshell protein | ABF13207.1 |
|  | *Homo sapiens* (Human) | Elastin | AAC98395.1 |
|  | *Drosophila melanogaster* (Fly) | Cuticular protein 47Ef | NP_001188905.1 |
|  | *Petromyzon marinus* (Lamprey) | Lamprin | P33575.1 |
|  | *Rattus norvegicus* (Rat) | Keratin | NP_001008802.2 |
|  | *Homo sapiens* (Human) | Loricrin | NP_000418.2 |
|  | *Homo sapiens* (Human) | Collagen | NP_000080.2 |
|  | *Oryza sativa* (Rice) | Glycine-rich cell wall protein | P0C5C7.1 |
|  | *Phaseolus vulgaris* (Stringbean) | Glycine-rich cell wall structural protein 1.0 | P10495.1 |
|  | *Phaseolus vulgaris* (Stringbean) | Glycine-rich cell wall structural protein 1.8 | P10496.1 |
|  | *Pinctada fucata* (Oyster) | Shematrin 1 | BAE93433.1 |
|  | *Pinctada fucata* (Oyster) | Shematrin 2 | BAE93434.1 |
|  | *Pinctada fucata* (Oyster) | Shematrin 3 | BAE93435.1 |
|  | *Pinctada fucata* (Oyster) | Shematrin 4 | BAE93436.1 |
|  | *Pinctada fucata* (Oyster) | Shematrin 5 | BAE93437.1 |
|  | *Pinctada fucata* (Oyster) | Shematrin 6 | BAE93438.1 |
|  | *Pinctada fucata* (Oyster) | Shematrin 7 | BAE93439.1 |
|  | *Haliotis rufescens* (Abalone) | Lustrin A | AAB95154.1 |
|  | *Pinctada fucata* (Oyster) | Nacrein | BAA11940.1 |
|  | *Pinctada fucata (Oyster)* | Pearlin | BAA75626.1 |
|  | *Bombus terrestris* (Bumblebee) | Silk fibrion | ABW21697.1 |
|  | *Bombus terrestris* (Bumblebee) | Silk fibrion | ABW21696.1 |
|  | *Bombus terrestris* (Bumblebee) | Silk fibrion | ABW21695.1 |
|  | *Bombus terrestris* (Bumblebee) | Silk fibrion | ABW21694.1 |
|  | *Myrmecia forficata* (Ant) | Silk fibrion | ABW21701.1 |
|  | *Myrmecia forficata* (Ant) | Silk fibrion | ABW21700.1 |
|  | *Myrmecia forficata* (Ant) | Silk fibrion | ABW21699.1 |
|  | *Myrmecia forficata* (Ant) | Silk fibrion | ABW21698.1 |
|  | *Oecophylla smaragdina* (Ant) | Silk fibrion | ABW21705.1 |
|  | *Oecophylla smaragdina* (Ant) | Silk fibrion | ABW21704.1 |
|  | *Oecophylla smaragdina* (Ant) | Silk fibrion | ABW21703.1 |
|  | *Oecophylla smaragdina* (Ant) | Silk fibrion | ABW21702.1 |
|  | *Apis mellifera* (Honeybee) | Silk fibrion 1 | NP_001129678.1 |
|  | *Apis mellifera* (Honeybee) | Silk fibrion 2 | NP_001129679.1 |
|  | *Apis mellifera* (Honeybee) | Silk fibrion 3 | NP_001129680.1 |
|  | *Apis mellifera* (Honeybee) | Silk fibrion 4 | NP_001129681.1 |
|  |  |  |  |
| **Non-silk-like** | *Strongylocentrotus purpuratus* (Urchin) | Hedgehog | NP_001012720.1 |
|  | *Arabidopsis thaliana* (Plant) | Glycine-rich protein 3 short isoform | AEC05924.1 |
|  | *Xenopus laevis* (Frog) | Exostosin 1 | AAO84329.1 |
|  | *Solanum lycopersicum* (Tomato) | Peptide N-glycanase | ACX94235.2 |
|  | *Homo sapiens* (Human) | Polydom | NP_699197.3 |
|  | *Artemia sinica* (Brine shrimp) | Apoptosis inhibitor protein 5 | AEG67303.1 |
|  | *Sus scrofa* (Pig) | N-acetylgalactosamine-6-sulfatase precursor | AAL55968.1 |
|  | *Danio rerio* (Zebrafish) | immunoglobulin binding protein | AAT68067.1 |
|  | *Ruditapes decussatus* (Clam) | complement component C3 | ACN37845.1 |
|  | *Homo sapiens* (Human) | carboxyl ester lipase | AAA51973.1 |
|  | *Halocynthia roretzi*  (Ascidian) | metalloproteinase | ACA03771.1 |
|  | *Rattus norvegicus* (Rat) | collagen prolyl 4-hydroxylase alpha III subunit | AAQ87605.1 |
|  | *Homo sapiens* (Human) | cadherin related 23 | AAG48303.1 |
|  | *Rattus norvegicus* (Rat) | Sodium channel, nonvoltage-gated | AAH70902.1 |
|  | *Crassostrea gigas* (Oyster) | LBP/BPI | AAN84552.1 |
|  | *Ostrinia nubilalis* (Corn borer) | peptidoglycan recognition protein A | ADU33184.1 |
|  | *Taenia pisiformis* (Platyhelmith) | cathepsin L-like cysteine protease | AEG19548.1 |
|  | *Schistocerca americana* (Locust) | lachesin | AAC37185.1 |
|  | *Cynoglossus semilaevis* (Sole) | follistatin | AEF32782.1 |
|  | *Rattus norvegicus* (Rat) | fibrinogen beta chain precursor | NP_064456.2 |
|  | *Mus musculus* (Mouse) | sialic acid binding immunoglobin-like lectin | ABO14788.1 |
|  | *Squalus acanthias* (Dogfish) | C-type natriuretic peptide receptor precursor | AAC78297.1 |
|  | *Botryllus schlosseri* (Ascidian) | rhamnose binding lectin isoform 1 | ABU23776.1 |
|  | *Homo sapiens* (Human) | scavenger receptor class F member 1 | NP_003684.2 |
|  | *Litopenaeus vannamei* (Prawn) | C-type lectin-1 | ADW08726.1 |
|  | *Penaeus monodon* (Prawn) | ribophorin I | ABU54836.1 |
|  | *Drosophila melanogaster* (Fruit fly) | amylase | AAQ82589.1 |
|  | *Canis lupus familiaris* (Dog) | Tyrosinase | AAQ17535.1 |
|  | *Euperipatoides rowelli* (Onychophoran) | Proline rich protein 2a (glue) | ADI48488.1 |
|  | *Euperipatoides rowelli* (Onychophoran) | Proline rich protein 2b (glue) | ADI48489.1 |
|  | *Euperipatoides rowelli* (Onychophoran) | Proline rich protein 3 (glue) | ADI48490.1 |
|  | *Bombyx mori* (Silkworm) | Sericin 1 (glue) | NP_001037506.1 |
|  | *Bombyx mori* (Silkworm) | Sericin 2 (glue) | NP_001166287.1 |
|  | *Bombyx mori* (Silkworm) | Sericin 3 (glue) | NP_001108116.1 |
|  | *Aplysia californica* (Sea hare) | GABA A receptor alpha subunit | AAL37252.1 |
|  | *Epinephelus coioides* (Grouper) | phospholipid hydroperoxide glutathione peroxidase | AEG78385.1 |
|  | *Saccoglossus kowalevskii* (Acorn worm) | R-spondin | ACY92631.1 |
|  | *Leucopsarion petersii* (Goby) | neuropeptide Y | BAK09590.1 |
|  | *Acropora millepora* (Coral) | galaxin | ADI50283.1 |
|  | *Penaeus monodon* (Prawn) | thrombospondin | ADK63101.1 |
|  | *Salmo salar* (Salmon) | Decorin | NP_001167033.1 |
|  | *Canis lupus familiaris* (Dog) | kallikrein-14 | NP_001159351.1 |
|  | *Drosophila melanogaster* (Fruitfly) | unpaired | AAC69620.1 |
|  | *Caenorhabditis elegans* (Nematode) | osteonectin/sparc | AAA16827.1 |
|  | *Xenopus laevis* (Frog) | glutamate carboxypeptidase | AAN86572.1 |
|  | *Homo sapiens* (Human) | WISP-3 | AAC96323.1 |
|  | *Capra hircus* (Goat) | parathyroid hormone-like protein | ADK36630.1 |
|  | *Oryctolagus cuniculus* (Rabbit) | retinoschisin | ABU55903.1 |
|  | *Rattus norvegicus* (Rat) | Insulin-like growth factor binding protein 7 | AAH86582.1 |
|  | *Xenopus laevis* (Frog) | Isthmin | AAM13976.1 |
|  | *Danio rerio* (Zebrafish) | EGF-like-domain multiple 7 | AAS99349.1 |
|  | *Drosophila melanogaster* (Fruit fly) | argos | AAA28379.1 |
|  | *Caenorhabditis elegans* (Nematode) | hemicentin | AAC26792.1 |
|  | *Homo sapiens* (Human) | follicular dendritic cell secreted peptide | AAN01116.1 |
|  | *Oryzias latipes* (Medaka) | osteoprotegerin | AEC32093.1 |
|  | *Rattus norvegicus* (Rat) | osteocrin | AAQ94967.1 |
|  | *Xenopus laevis* (Frog) | Xerl | BAA95001.1 |
|  | *Mus musculus* (Mouse) | ran-binding protein 10 | NP_665823.2 |
|  | *Heterodera glycines* (Nematode) | peptidylglycine alpha-hydroxylating monooxygenase | AAO92288.2 |
|  | *Paralichthys olivaceus* (Flounder) | serotransferrin | ACZ92269.1 |
|  | *Drosophila melanogaster* (Fruit fly) | kekkon5 | AAO43730.1 |
|  | *Rattus norvegicus* (Rat) | ADAMTS5 | AAQ88212.1 |
|  | *Drosophila melanogaster* (Fruit fly) | Papilin | AAG37995.1 |
|  | *Cyprinus carpio* (Carp) | Fetuin | AAO74861.1 |
|  | *Haliotis discus discus* (Abalone) | incilarin A | ABO26661.1 |
|  | *Aplysia californica* (Sea hare) | Pedal peptide | AAP57098.1 |
|  | *Saccharomyces cerevisiae* (Yeast) | Hem4p | NP_014921.1 |
|  | *Canis lupus familiaris* (Dog) | angiopoietin | BAD54826.1 |
|  | *Manduca sexta* (Moth) | lacunin | AAF04457.1 |
|  | *Homarus americanus* (Lobster) | prepro-myosuppressin | ACX46385.1 |
|  | *Homo sapiens* (Human) | melanoma chondroitin sulfate proteoglycan | AAQ62842.1 |
|  | *Ornithodoros parkeri* (Tick) | fibrinogen domain-containing salivary secreted protein | ABR23368.1 |
|  | *Hordeum vulgare* (Barley) | purple acid phosphatase isoform a | AEG77016.1 |
|  | *Canis lupus familiaris* (Dog) | cubilin | AAF14258.1 |
|  | *Mus musculus* (Mouse) | gamma-glutamyl leukotrienase | AAC71001.1 |
|  | *Lymnaea stagnalis* (Pond snail) | alpha-amidating enzyme precursor 2 | AAD42259.1 |
|  | *Pacifastacus leniusculus* (Crayfish) | pacifastin heavy chain precursor | AAC64660.1 |
|  | *Caenorhabditis elegans* (Nematode) | GLY8 | AAC13678.1 |
|  | *Stylophora pistillata* (Coral) | carbonic anhydrase | ACE95141.1 |
|  | *Aplysia californica* (Sea hare) | temptin | AAP73787.1 |
|  | *Mus musculus* (Mouse) | acetylcholine receptor subunit delta precursor | NP_067611.2 |
|  | *Homo sapiens* (Human) | properdin precursor | NP_002612.1 |
|  | *Epinephelus coioides* (Grouper) | POMC | AAO11696.1 |
|  | *Ornithodoros parkeri* (Tick) | salivary lipocalin | ABR23385.1 |
|  | *Homo sapiens* (Human) | tripeptidyl-peptidase 1 preproprotein | NP_000382.3 |
|  | *Homo sapiens* (Human) | fatty-acid amide hydrolase 1 | NP_001432.2 |
|  | *Oryctolagus cuniculus* (Rabbit) | blood coagulation factor X | AAB62542.1 |
|  | *Cavia porcellus* (Guinea pig) | interferon-gamma | AAN75515.1 |
|  | *Branchiostoma floridae* (Amphioxus) | amphiDkk3 | AEG80153.1 |
|  | *Homo sapiens* (Human) | prolactin receptor | NP_001191247.1 |
|  | *Xenopus laevis* (Frog) | norrin | ACD44936.1 |
|  | *Danio rerio* (Zebrafish) | notum3 | ACH92954.1 |
|  | *Hypocrea virens* (Fungi) | Mrsp1 | ABJ80893.1 |
|  | *Dictyostelium discoideum* (Slime mould) | AprA | AAU95081.1 |
|  | *Paracoccidioides brasiliensis* (Fungi) | aspartyl proteinase | AAP32823.1 |
|  | *Rattus norvegicus* (Rat) | gliomedin | AAP22419.1 |
|  | *Danio rerio* (Zebrafish) | southpaw | AAP22500.1 |
|  | *Xenopus laevis* (Frog) | gremlin | AAC41279.1 |
|  | *Solanum lycopersicum* (Tomato) | Lemir | AAC63057.1 |
|  | *Gallus gallus* (Chicken) | zona pellucida sperm-binding protein 3 | NP_989720.2 |
