# Supplementary material for: The Widespread Prevalence and Functional Significance of Silk-Like Structural Proteins in Metazoan Biological Materials
Source: PLoS One. 2016 Jul 14;11(7):e0159128. doi: 10.1371/journal.pone.0159128 (PMC4944945; doi:10.1371/journal.pone.0159128)
Supplement: S2 Table — Table of known silkworm silk-like proteins and their accession numbers. (DOCX) [file pone.0159128.s006.docx]

**S2 Table: Known *B. mori* silk-like proteins**

| **Sequence name** | **Genbank accession** |
| --- | --- |
| Fibroin heavy chain | NP_001106733.1 |
| Chorion class CA ERA1 | NP_001112376.1 |
| Chorion class CA ERA2 | NP_001112377.1 |
| Chorion class CA ERA3 | NP_001112378.1 |
| Chorion class CA ERA4 | NP_001112373.1 |
| Chorion class CA ERA5 | NP_001112379.1 |
| Chorion protein ERB1 | NP_001108477.1 |
| Chorion protein ERB2 | NP_001112380.1 |
| Chorion protein ERB3 | NP_001112381.1 |
| Chorion class B ERB4 | NP_001112382.1 |
| Chorion protein ERB5 | NP_001112383.1 |
| Chorion class CB M5H4 | NP_001112374.1 |
