# Supplementary material for: The Widespread Prevalence and Functional Significance of Silk-Like Structural Proteins in Metazoan Biological Materials
Source: PLoS One. 2016 Jul 14;11(7):e0159128. doi: 10.1371/journal.pone.0159128 (PMC4944945; doi:10.1371/journal.pone.0159128)
Supplement: S3 Table — Table with details of the data used for analyses, including websites and references. (DOCX) [file pone.0159128.s007.docx]

**S3 Table: Sources of sequence data used for analysis**

| **Organism** | **Common name** | **Type** | **Details** | **Source** | **Accessed** | **Ref** |
| --- | --- | --- | --- | --- | --- | --- |
| *Homo sapiens* | Human | Genome | Translation of all known gene models from hg19 | http://hgdownload.cse.ucsc.edu/goldenPath/hg19/database/knownGenePep.txt.gz | 20-Dec-13 | (1) |
| *Gallus gallus* | Chicken | Genome | All known and novel gene models 'pep' | ftp://ftp.ensembl.org/pub/release-74/fasta/gallus_gallus/pep/ | 19-Dec-13 | (2) |
| *Strongylocentrotus purpuratus* | Sea urchin | Genome | Gnomon proteins | http://www.spbase.org/SpBase/downloads.php | 9-Oct-12 | (3) |
| *Pinctada maxima* | Pearl oyster | EST | From juvenile and adult mantle | n/a | n/a | (4) |
| *Haliotis asinina* | Abalone | EST | Adult mantle | n/a | n/a | (5) |
| *Capitella teleta* | Polychaete worm | Genome | Best gene models | http://genome.jgi.doe.gov/Capca1/Capca1.download.ftp.html | 18-Dec-13 | (6) |
| *Caenorhabditis elegans* | Nematode | Genome | Current predicted proteins | ftp://ftp.wormbase.org/pub/wormbase/species/c_elegans/sequence/protein/ | 15-Jan-13 | (7) |
| *Tribolium castaneum* | Beetle | Genome | Glean proteins Glean.prot.51906.fa | ftp://ftp.bioinformatics.ksu.edu/pub/BeetleBase/latest/Sequences/GLEAN/ | 7-Jan-14 | (8) |
| *Bombyx mori* | Silkworm | Protein | NCBI protein database | http://www.ncbi.nlm.nih.gov | 7-Jan-13 | n/a |
| *Nematostella vectensis* | Anemone | Genome | Best gene models | http://genome.jgi-psf.org/Nemve1/Nemve1.download.ftp.html | 27-Jun-11 | (9) |
| *Acropora digitifera* | Coral | Genome | Predicted proteins - assembly version 1 | http://marinegenomics.oist.jp/acropora_digitifera/download | 12-Dec-11 | (10) |
| *Trichoplax adhaerens* | Placozoan | Genome | Best gene models from JGI | http://genome.jgi-psf.org/Triad1/Triad1.download.ftp.html | 24-Jun-11 | (11) |
| *Leucetta chagosensis* | Calcareous sponge | EST | ESTs from NCBI | http://www.ncbi.nlm.nih.gov | 7-Dec-11 | n/a |
| *Amphimedon queenslandica* | Siliceous sponge | Genome | Predicted proteins – Aq1.pep | ftp://ftp.jgi-psf.org/pub/JGI_data/  Amphimedon_queenslandica/annotation/ | 21-Jul-11 | (12) |

1. Rosenbloom KR*, et al.* (2013) ENCODE data in the UCSC Genome Browser: year 5 update. *Nucleic Acids Res* 41(Database issue):D56-63.

2. Flicek P*, et al.* (2013) Ensembl 2013. *Nucleic Acids Res* 41(Database issue):D48-55.

3. Cameron RA, Samanta M, Yuan A, He D, Davidson E (2009) SpBase: the sea urchin genome database and web site. *Nucleic Acids Res* 37(Database issue):D750-754.

4. McDougall C, Aguilera F, Degnan BM (2013) Rapid evolution of pearl oyster shell matrix proteins with repetitive, low-complexity domains. *J R Soc Interface* 10(82):20130041.

5. Jackson DJ*, et al.* (2010) Parallel evolution of nacre building gene sets in molluscs. *Mol Biol Evol* 27(3):591-608.

6. Simakov O*, et al.* (2013) Insights into bilaterian evolution from three spiralian genomes. *Nature* 493(7433):526-531.

7. Yook K*, et al.* (2012) WormBase 2012: more genomes, more data, new website. *Nucleic Acids Res* 40(Database issue):D735-741.

8. Kim HS*, et al.* (2010) BeetleBase in 2010: revisions to provide comprehensive genomic information for *Tribolium castaneum*. *Nucleic Acids Res* 38(Database issue):D437-442.

9. Putnam NH*, et al.* (2007) Sea anemone genome reveals ancestral eumetazoan gene repertoire and genomic organization. *Science* 317(5834):86-94.

10. Shinzato C*, et al.* (2011) Using the *Acropora digitifera* genome to understand coral responses to environmental change. *Nature* 476(7360):320-323.

11. Srivastava M*, et al.* (2008) The *Trichoplax* genome and the nature of placozoans. *Nature* 454(7207):955-960.

12. Srivastava M*, et al.* (2010) The *Amphimedon queenslandica* genome and the evolution of animal complexity. *Nature* 466(7307):720-726.
